# Supplementary material for: MetaRibo-Seq measures translation in microbiomes
Source: Nat Commun. 2020 Jun 29;11:3268. doi: 10.1038/s41467-020-17081-z (PMC7324362; doi:10.1038/s41467-020-17081-z)
Supplement: Supplementary file 10 — Supplementary Data 7 [file 41467_2020_17081_MOESM10_ESM.zip › File2/Confidence_High_Taxonomy/433304_out.krona.html]

Javascript must be enabled to view this page.

members
magnitude
magnitudeUnassigned
count
unassigned
taxon
rank

433304\_out

10

superkingdom
2
10

phylum
10
976

10
200643
class

171549
10
order

815
10
family

816
10
1

SRS147727\_contig\_number\_contig-100\_200.201
genus

species

SRS076804\_contig\_number\_21268SRS104311\_contig\_number\_40479
2
2292956

2292955
1
species

SRS022609\_contig\_number\_49535

6
28116

SRS013521\_contig\_number\_13298SRS022713\_contig\_number\_5574SRS024132\_contig\_number\_49858SRS042628\_contig\_number\_33187SRS049995\_contig\_number\_35688SRS143895\_contig\_number\_29733
species
